# Population structure of a widespread bat (*Tadarida brasiliensis*) in an island system

**DOI:** 10.1002/ece3.3233

**Published:** 2017-08-17

**Authors:** Kelly A. Speer, Brandi Jo Petronio, Nancy B. Simmons, Rebecca Richey, Kristin Magrini, J. Angel Soto‐Centeno, David L. Reed

**Affiliations:** ^1^ Department of Biology University of Florida Gainesville FL USA; ^2^ Florida Museum of Natural History Gainesville FL USA; ^3^ Richard Gilder Graduate School American Museum of Natural History New York NY USA; ^4^ Division of Vertebrate Zoology Department of Mammalogy American Museum of Natural History New York NY USA

**Keywords:** Bahamas, Caribbean, Chiroptera, dispersal, Molossidae, West Indies

## Abstract

Dispersal is a driving factor in the creation and maintenance of biodiversity, yet little is known about the effects of habitat variation and geography on dispersal and population connectivity in most mammalian groups. Bats of the family Molossidae are fast‐flying mammals thought to have potentially high dispersal ability, and recent studies have indicated gene flow across hundreds of kilometers in continental North American populations of the Brazilian free‐tailed bat, *Tadarida brasiliensis*. We examined the population genetics, phylogeography, and morphology of this species in Florida and across islands of The Bahamas, which are part of an island archipelago in the West Indies. Previous studies indicate that bats in the family Phyllostomidae, which are possibly less mobile than members of the family Molossidae, exhibit population structuring across The Bahamas. We hypothesized that *T. brasiliensis* would show high population connectivity throughout the islands and that *T. brasiliensis* would show higher connectivity than two species of phyllostomid bats that have been previously examined in The Bahamas. Contrary to our predictions, *T. brasiliensis* shows high population structure between two groups of islands in The Bahamas, similar to the structure exhibited by one species of phyllostomid bat. Phylogenetic and morphological analyses suggest that this structure may be the result of ancient divergence between two populations of *T. brasiliensis* that subsequently came into contact in The Bahamas. Our findings additionally suggest that there may be cryptic species within *T. brasiliensis* in The Bahamas and the West Indies more broadly.

## 1 INTRODUCTION

As the only mammals capable of powered flight, bats are unique organisms for the study of movement in fragmented habitats. Many mammals undertake long‐distance dispersal events during natal dispersal (Sutherland, Harestad, Price, & Lertzman, 2000) or seasonal migrations (Berger, 2004), but the vagility of bats allows them to colonize even isolated oceanic islands such as Hawaii and the Galapagos Islands (McCracken, Hayes, Cevallos, Guffey, & Romero, 1997; Russell, Pinzari, Vonhof, Olival, & Bonaccorso, 2015; Ziegler, Howarth, & Simmons, 2016). As dispersal is a key component in the evolution of populations (Moussy et al., 2013), it is important to understand barriers that impact the movement of the most mobile mammals.

Previous research on island bats suggests that despite their mobility, open water between islands (or between islands and the mainland) may pose a significant barrier to dispersal of many species (Bonaccorso & Mcguire, 2013; Carstens, Sullivan, Davalos, Larsen, & Pedersen, 2004; Fleming, Murray, & Carstens, 2010; Moussy et al., 2013; Muscarella, Murray, Ortt, Russell, & Fleming, 2011; Salgueiro, Coelho, Palmeirim, & Ruedi, 2004). Phylogeographic studies of West Indian bats belonging to the family Phyllostomidae have shown that populations of *Ardops nichollsi*,* Erophylla sezekorni*, and *Macrotus waterhousii* are structured between islands (Carstens et al., 2004; Muscarella et al., 2011). *Ardops nichollsi* and *Erophylla sezekorni* are island endemics with highly restricted geographic ranges and were expected to exhibit low population connectivity between islands. However, Muscarella et al. (2011) found that populations of *E. sezekorni* are less structured than populations of *Macrotus waterhousii*, which has a broader range. Not all species exhibit population structuring between islands. *Artibeus jamaicensis* and *Brachyphylla cavernarum* show high population connectivity between islands (Carstens et al., 2004; Larsen et al., 2007). These findings indicate that factors influencing population structure in West Indian bats are complex and range size and migratory ability (all of the above‐mentioned species are nonmigratory) may not be good predictors of connectivity in this system.

All previous studies of population structure in the West Indies have examined members of the family Phyllostomidae (i.e., Carstens et al., 2004; Larsen et al., 2007; Muscarella et al., 2011). Phyllostomid bats in the West Indies tend to have wings with low to average aspect ratios, average to high wing loading, and low to average wing tip indices (Jennings, Parsons, Barlow, & Gannon, 2004). These traits are characteristic of slow and maneuverable flight, and taxa with this wing morphology do not exhibit fast flight in open spaces (Jennings et al., 2004; Norberg & Rayner, 1987). In contrast, bats in the family Molossidae typically have wings with high aspect ratio, high wing loading, and average wing tip indices (Norberg & Rayner, 1987). These characteristics are associated with fast flight often above the canopy, inability to maneuver in clutter, high dispersal ability, and low population genetic structure (Burns & Broders, 2014; Norberg & Rayner, 1987; Taylor, Goodman, Schoeman, Ratrimomanarivo, & Lamb, 2012). If wing morphology and flight pattern are correlated with dispersal across open water, then we expect phyllostomid bats to show less population connectivity between islands than molossid bats.

To better understand the role of open water barriers in structuring bat diversity, we examined the population connectivity of a molossid bat, *Tadarida brasiliensis*, in the West Indies. *Tadarida brasiliensis* is widespread from North America through Central America and South America and is also common on islands of the West Indies (Simmons, 2005). In temperate portions of its range, *T. brasiliensis* undergoes seasonal migrations of 1,800 km (Cockrum, 1969; Glass, 1982). Bats of this species regularly fly more than 50 km in a single night while foraging and have been documented flying at high altitudes of more than 3,000 m (the highest altitude recorded for feeding calls is 862 m; Williams, Ireland, & Williams, 1973; Best & Geluso, 2003; McCracken et al., 2008). These observations are consistent with the hypothesis that *T. brasiliensis* will be better able to move across open water between islands in the West Indies and will exhibit less population structure than observed previously in phyllostomid species.

The West Indies, like many island systems, is well suited for phylogeographic studies of terrestrial organisms because islands form discrete fragments of suitable habitat, and these islands are isolated enough from the mainland to allow high endemism (Whittaker & Fernandez‐Palacios, 2007: Chapter 1; Ricklefs & Bermingham, 2008; Hedges, 2001). Islands of the West Indies (The Bahamas, Greater and Lesser Antilles, and Turks and Caicos Islands) have varied geologic histories and include a range of island area and habitat types (dry tropical to wet tropical; Ricklefs & Bermingham, 2008). Terrestrial vertebrates colonized the West Indies by dispersal over water or across land bridges (one possible exception being frogs in the genus *Eleutherodactylus*, which may have vicariantly colonized the West Indies) from North America, Central America, and South America (Hedges, 1996, 2006).

**Figure 1:**
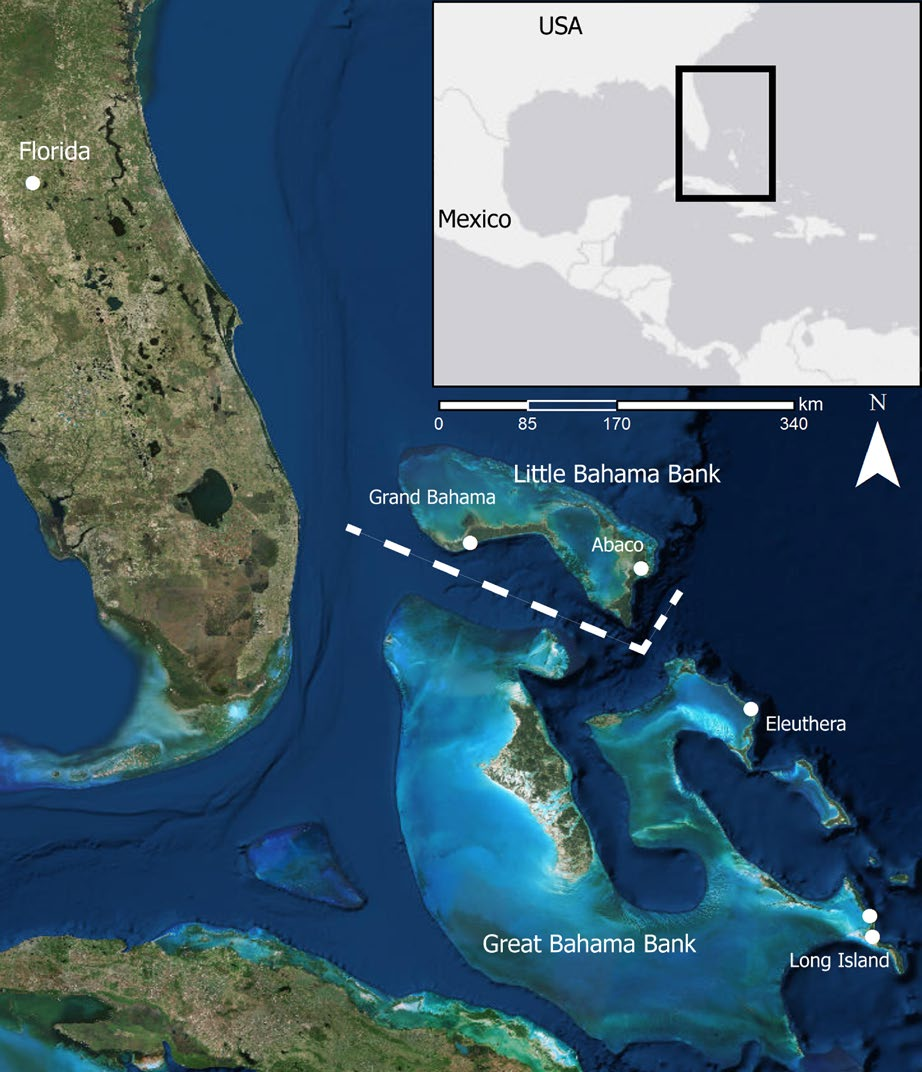
Map showing sampling localities (white dots) from which *T. brasiliensis* was collected in The Bahamas. Banks of islands are labeled along with the Northwest and Northeast Providence Channels (NPC, dashed line)

The northernmost islands of the West Indies are The Bahamas, an archipelago of 29 islands and over 600 cays (small islets) located north of Cuba and east of Florida between 20° and 28°N. The Bahamas is generally divided into six major banks that each form island groups (Figure 1) including Little Bahama Bank (Abaco Islands and Grand Bahama Island), Great Bahama Bank (Andros Island, Bimini, Berry Islands, Cat Island, Eleuthera, Exuma Islands, Long Island, New Providence, and Ragged Islands), Crooked and Acklins Islands, Great and Little Inagua, Mayaguana, and San Salvador. These banks are shallowly submerged carbonate platforms that are separated by deep oceanic trenches (Carew & Mylroie, 2004). It is not known when terrestrial vertebrates colonized The Bahamas, but exposed landmass of these islands was formed in the Pleistocene and Holocene and the fossil record of The Bahamas does not currently predate the Pleistocene (Carew & Mylroie, 2004; Morgan & Woods, 1986; Soto‐Centeno & Steadman, 2015; Steadman et al., 2015). This suggests that terrestrial vertebrates colonized The Bahamas during the Pleistocene (2.5 ma–11.7 ka) and possibly not until more recently than 120 ka due to a greater than 50% reduction in island size during the last interglacial sea‐level high stand (Carew & Mylroie, 2004; Morgan, 2001). As a result, species diversity and endemism in The Bahamas are low compared to the Greater Antilles, and species composition is strongly affected by source communities on nearby islands (especially Cuba) and the North American mainland (Florida; Morgan, 1989; Speer et al., 2015).

Our knowledge of bat dispersal among islands of The Bahamas is limited, with only one study of West Indian bat phylogeography including samples from The Bahamas. This study found that populations of two phyllostomid bats, *Erophylla sezekorni* and *Macrotus waterhousii*, were distinct between the Great Bahama Bank and Little Bahama Bank, which are separated by the Northwest and Northeast Providence Channels (hereafter referenced together as NPC; Muscarella et al., 2011). To examine the potential effect of wing morphology and correlated dispersal ability on population connectivity across the NPC, we estimated gene flow in *Tadarida brasiliensis*, a molossid bat. We used nuclear microsatellites to examine gene flow and recent population connectivity. To assess deeper time patterns of gene flow between island and mainland populations, we used mitochondrial DNA. We also gathered morphological data to assess congruence with sequence and microsatellite data. This study examines phylogeography in the West Indies by examining dispersal and the potential influence of water barriers on molossid bats.

## 2 MATERIALS AND METHODS

### 2.1 Sampling

We collected organ tissue or wing punches from 89 individuals of *Tadarida brasiliensis* in the spring and summer of 2013 and 2014 and loaned tissues and/or skeletons of 80 individuals from the Florida Museum of Natural History, totaling 139 individuals examined genetically and 65 individuals examined morphologically. These samples were from Florida (*n* = 30) and four islands of The Bahamas spanning the NPC: Abaco (*n* = 22) and Grand Bahama (*n* = 30) on the Little Bahama Bank, and Eleuthera (*n* = 40) and Long Island (*n* = 50) on the Great Bahama Bank (Figure 1; Appendix S1). While some temperate populations of *T. brasiliensis* are migratory, there is no evidence that tropical populations undergo migration (Morales, Villalobos, Velazco, Simmons, & Piñero, 2016). *T. brasiliensis* is common in The Bahamas, and bats sampled in this study were collected from caves and abandoned buildings. Individuals collected from day roosts were captured using hand nets. In Grand Bahama, we used mist nets to capture bats from underneath the entryway of an abandoned hotel because adults were clustered with pups. As *T. brasiliensis* females do not carry their pups with them (Gustin & McCracken, 1987), mist nets provided a means to safely sample the adult population without harming the pups, which were too young to fly. In Long Island, we were able to collect samples from two caves (25 individuals sampled from each cave including those loaned from the Florida Museum of Natural History) approximately 40 km away from each other to test within‐island dispersal. Interestingly, *T. brasiliensis* was found only in abandoned buildings on islands of the Little Bahama Bank and only in caves on islands of the Great Bahama Bank. There are caves inhabited by other bat species in the Little Bahama Bank (Speer et al., 2015), but these caves may not be suitable for *T. brasiliensis*. For each individual captured, we recorded the sex, reproductive status, body mass (g), forearm length (mm), hind foot length (mm), ear length (mm), and tragus length (mm) in the field. All animal capture and handling procedures were approved by the University of Florida Institutional Animal Care and Use Committee (#201004427) and followed guidelines set by the American Society of Mammalogists (Sikes, Gannon, & the Animal Care and Use Committee of the American Society of Mammalogists, 2011).

Sterile biopsy punches (Acuderm Inc., Ft. Lauderdale, FL, USA), 4 mm in diameter, were used to collect wing punches. In most cases, two wing punches were collected from each individual, one punch from each wing. Halothane (Sigma‐Aldrich, St. Louis, MO, USA) was used to euthanize voucher specimens that were collected, and heart, kidney, liver, lung, and spleen tissue was taken from these specimens. Organ tissue and wing punches were preserved in 95% ethanol at room temp for no more than 2 weeks prior to being stored at −20°C for the duration of laboratory work. All tissues were deposited at the Florida Museum of Natural History (Appendix S1). Voucher specimens were deposited at the Florida Museum of Natural History (Appendix S1) and the National Museum of The Bahamas, The Antiquities, Monuments, and Museums Corporation.

### 2.2 DNA extraction

DNA was extracted from organ tissue (normally liver) or wing punches using QIAamp DNA Micro Kits (Qiagen, Inc., Valencia, CA, USA) and ZR Genomic DNA Tissue MicroPrep kits (Zymo Research, Irvine, CA, USA) following manufacturer's instructions. Elution buffer was briefly heated prior to pipetting it onto the spin columns to improve DNA yield. Wing punches were cut in half prior to extractions. In the case of tissue samples, approximately 50 mg of organ tissue was used for extractions. Gel electrophoresis was used to verify the success of extractions and PCRs (see Supporting Information).

### 2.3 Microsatellite data

We genotyped 136 individuals of *Tadarida brasiliensis* at nine microsatellite loci (TabrA10, TabrA30, TabrD10, TabrD15, TabrE9, TabrH2, TabrH3, TabrH6, and TabrH12) using primers developed by Russell, Turmelle, Brown, and McCracken (2005). We adapted these primers for use in a three‐primer approach or nested PCR using universal tags to fluorescently label PCR products (Schuelke, 2000). Fluorescently labeled universal primers are more cost‐effective than using dye‐labeled locus‐specific primers (Blacket, Robin, Good, & Lee, 2012). We followed the dye‐labeling scheme outlined in Ascunce et al. (2013). Microsatellites were amplified individually using two procedures. Procedure 1 used 15 μl reaction volume containing final concentrations of 1.25× master mix (2.5× HotMasterMix, 5 PRIME, Inc. Gaithersburg, MD, USA); 0.03–0.10 μmol/L unlabeled forward primer with universal tail (Integrated DNA Technologies, Inc., Coralville, IA, USA); 0.33–0.67 μmol/L unlabeled reverse primer (Integrated DNA Technologies, Inc.); 0.33–0.67 μmol/L fluorescently labeled universal primer (Applied Biosystems, Foster City, CA, USA); 1 μl total genomic DNA (concentrations of DNA extractions not quantified). Procedure 2 uses Type‐it Microsatellite PCR Master Mix (Qiagen, Inc., Hilden, Germany) and 0.04 μmol/L final concentration of unlabeled forward primer with tail, with the rest of the protocol the same as in Procedure 1. We used Procedure 1 for most PCRs, but Procedure 2 was used for samples and loci (TabrE9 and TabrH3) that were difficult to amplify. Thermocycler protocols are the same for all primers and procedures, following Ascunce et al. (2013, protocol v2), and 50°C was used as the annealing temperature. Using four different dyes (6‐FAM, VIC, NED, and PET) allowed us to multiplex individuals prior to analysis by capillary separation. In most cases, PCR products were 4‐plexed, but when the ranges of expected amplicon lengths of two loci were more than 100 bp apart, we were able to multiplex further (up to 8‐plex). PCR products were diluted during multiplexing, by combining 1.5 μl of products containing 6‐FAM or VIC fluorophores, 3.0 μl of products containing NED or PET fluorophores, and adding sdH_2_O to reach a final volume of 50 μl. Genotyping was performed by capillary separation on the ABI3730xl DNA Analyzer at the Interdisciplinary Center of Biotechnology Research (ICBR) Genotyping Core at the University of Florida. Genescan 600 was used as the size standard for most genotyping procedures, but for TABR H2, Genescan 1200 was used. Allele calls are provided in Dryad (doi:10.5061/dryad.9n0p1).

### 2.4 Mitochondrial DNA data

We amplified the mitochondrial cytochrome *b* (751‐1,140 bases; cytb) for 45 individuals using primers MTCB‐F and MTCB‐R (Naidu, Fitak, Vega, & Culver, 2012; GenBank accession MF135735–MF135779). We used 15 μl reaction volumes containing final concentrations of 1.25× master mix (2.5× HotMasterMix, 5 PRIME, Inc.), 0.33 μmol/L forward primer, 0.33 μmol/L reverse primer, and 1 μl genomic DNA (concentration of DNA extractions was not quantified). We repeated PCRs that failed the first time using 20 μl reaction volume containing final concentrations of 1 × 5 PRIME Master Mix, 0.25 μmol/L of each the forward and reverse primers, and 1 μl genomic DNA. Thermocycler conditions followed Naidu et al. (2012). Successful PCRs were purified using 1 μl ExoSAP‐IT (Affymetrix, Inc., Santa Clara, CA, USA) for every 5 μl PCR product. Thermocycler conditions for the purification step followed manufacturer instructions. Following PCR purification, samples were sent to ICBR DNA Sequencing Core, where ABI Prism BigDye Terminator (part number 4336921, Applied Biosystems, Perkin‐Elmer Corp.) cycle sequencing reactions were performed. ABI prism BigDye Terminator v3.1 cycle sequencing reactions were performed in 10 μl reactions containing 10–20 ng of purified PCR product, 3.5 pmol/L of primer, 1 μl of BigDyeTM terminator, and 2 μl of 5× sequencing reaction buffer. Thermocycler protocols for the cycle sequencing reaction followed manufacturer instructions. Cycle sequencing products were purified using standard ethanol precipitation and were dried in a SpeedVac (ThermoSavant, Holbrook, NY, USA). Purified PCR products were suspended in Hi‐di formamide and then analyzed on an Applied Biosystems 3730xl Genetic Analyzer using POP‐7 sieving matrix and 1× capillary buffer.

### 2.5 Morphological data

**Figure 2:**
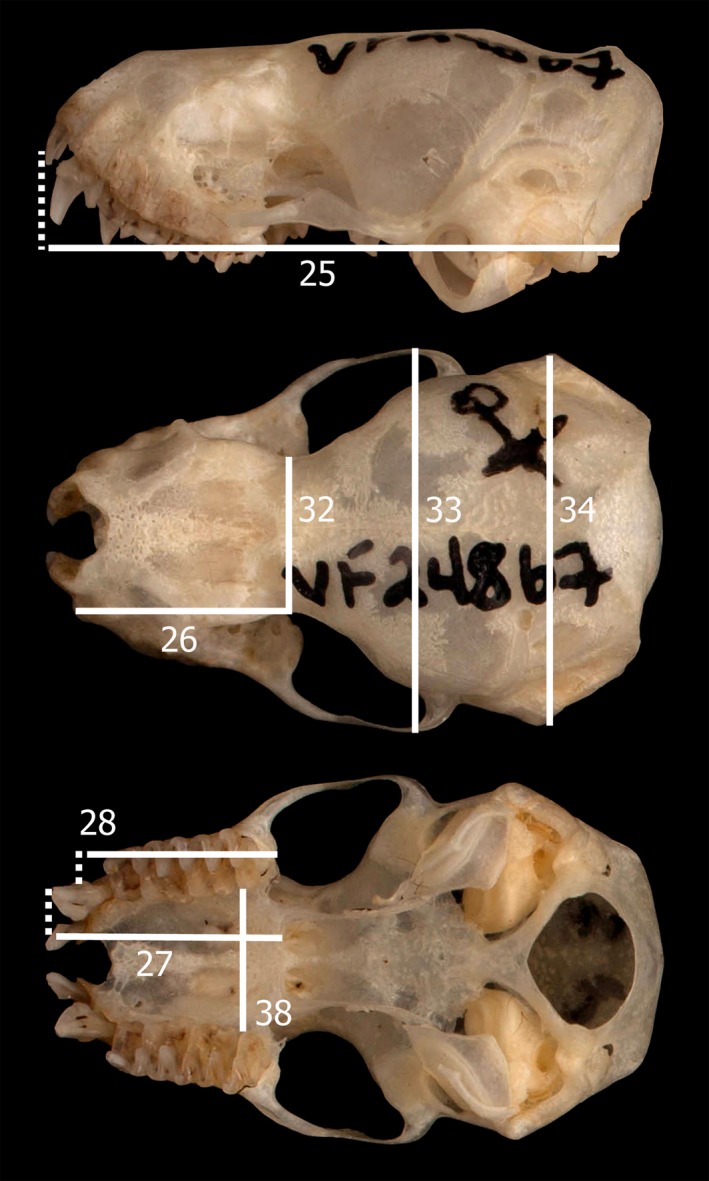
Skull measurements collected for this study are indicated with white lines, and numbers correspond with those in Freeman (1981)

We recorded forearm length and eight linear measurements of the skull from 65 specimens of *Tadarida brasiliensis* (20 from Florida, 15 from Little Bahama Bank, 30 from Great Bahama Bank; Appendix S1) using Pittsburgh digital calipers accurate to 0.01 mm and a stereo microscope (Leica S8 APO, Leica Microsystems, Inc., Buffalo Grove, IL, USA) outfitted with a 150‐Watt fiber optic illuminator (Techniquip, Micro Optics of Florida, Inc., Davie, FL, USA). Each measurement was taken three times and averaged to account for variation. Of the 65 individuals measured, 27 were also used in genetic analyses (Appendix S1). These measurements, a subset of those used by Freeman (1981), are condyloincisive length, rostral length, postorbital width, zygomatic breadth, breadth at mastoids, palatal length, maxillary tooth row length, and width of palate at upper molars (see Figure 2; measurements provided in Dryad, doi: 10.5061/dryad.9n0p1). We used R Studio (R Core Team 2015) to perform principal components analysis, using the stats and ggbiplot packages (Vu, 2011) and discriminant function analysis using the mass package (Venables & Ripley, 2002). Two‐tailed *t* tests showed no significant difference between males and females. However, our sample size for females was low, so we completed two sets of morphological analyses, one using the total dataset (males + females) and another using a male‐only dataset. Based on the results of population genetic analyses, the Little Bahama Bank and Florida were treated as a single locality separate from the Great Bahama Bank in all analyses.

### 2.6 Population differentiation

Raw microsatellite peak data were visualized and scored using Geneious v.6.1.7 (Kearse et al., 2012) with the microsatellite plugin. Deviations from Hardy–Weinberg equilibrium and the probability of heterozygote excess and heterozygote deficiency were calculated using exact tests implemented in GenePop v4.3 (Rousset, 2008). For all tests, the MCMC algorithm was used to calculate a *p*‐value for each locus in each population with default values of dememorization and batch number, and 100k permutations of the data for each batch. GenAlEx v6.501 (Peakall & Smouse, 2006; Peakall & Smouse, 2012) was used to calculate summary statistics and *F*_ST_. When examining highly polymorphic loci, the maximum value of *F*_ST_ may not reach 1 even when populations have no shared alleles (Jost, 2008). To overcome this issue, we also calculated Jost's D, which is based on the effective number of alleles rather than heterozygosity (Jost, 2008; Meirmans & Van Tienderen, 2004), and *G*”_ST_, which is standardized by the expected heterozygosity and corrected for sampling a small number of populations (Meirmans & Hedrick, 2011).

We used Structure v.2.3.4 (Pritchard, Stephens, & Donnelly, 2000) to assign individuals to populations under an admixture model with correlated allele frequencies, which assumes that individuals from different populations inherited a portion of their genome from a shared common ancestor (Falush, Stephens, & Pritchard, 2003). The dataset was run for five iterations at each *k*‐value ranging from 1 to 10. Each iteration was run for 50 k generations after a 5 k generation burn‐in. Convergence of the runs was verified using plots of log(Alpha) by iteration and lnL(K) by iteration. Results of the Structure analyses were visualized using Structure Harvester v0.6.94 (Earl & vonHoldt, 2011), which uses the Evanno method to assess the most likely number of populations (Evanno, Regnaut, & Goudet, 2005). In order to assess the Structure results under an assumption of *k* = 1, we used mean lnL(K) (see Supporting Information).

To test the correlation of distance with population structure, we measured the shortest geodesic distance between all pairs of islands and between all islands and the closest coastal point of Florida. These are the minimum possible distances required for an individual to disperse to reach each sampled location. We did not count the two localities on Long Island as distinct, because Structure indicated no population differentiation between these sites. We log‐transformed the genetic distances and geodesic distances and used a Mantel test implemented in GenAlEx (Peakall & Smouse, 2006; Peakall & Smouse 2012) to measure the correlation between pairwise genetic distance (*G*”_ST_) and pairwise geodesic distance, and used 999 random permutations to compare our findings to a random dataset.

### 2.7 Estimates of migration

We used BayesAss v3.0.3 (Wilson & Rannala, 2003) to measure contemporary (within the last two generations) migration rate between the Great and Little Bahama Banks. Individuals collected from Florida were excluded from this analysis, because of small sample size for that population. Treating each of the four islands as putative populations, we ran BayesAss for 50 million generations with a burn‐in of 10 million, sampling every 1,000 generations. The step sizes of migration rates were adjusted to *m* = 0.5, allele frequencies to *a* = 0.6, and inbreeding coefficients to *f* = 0.6, so that acceptance rate of proposed changes to these parameters remained between 20 and 60% through the duration of the run, in accordance with recommendations from the BayesAss manual. BayesAss was also run on a dataset where individuals from the Little Bahama Bank and Great Bahama Bank were grouped into two populations a priori. This grouped analysis was run for 10 million generations with a burn‐in of 2 million, sampling every 1,000 generations. Mixing parameters were adjusted for allele frequencies to *a* = 0.2 and inbreeding coefficients to *f* = 0.2 (see BayesAss manual). We used Tracer v1.6.0 (Rambaut, Suchard, Xie, & Drummond, 2014) to visualize that sampled parameter values through generations were mixing adequately and had converged prior to the end of the burn‐in in both the ungrouped and grouped dataset. In addition to estimating migration rate between populations, BayesAss also provides the posterior probability of the migrant history of each individual.

To test hypotheses of migration history, we calculated Bayes factors from the marginal likelihoods calculated in Migrate‐n v.3.3.0 (Beerli, 2006; Beerli & Palczewski, 2010). We tested four possible models of migration history: (1) varying migration rates between the Little Bahama Bank + Florida (northern population) and the Great Bahama Bank (southern population), (2) one panmictic population encompassing the northern and southern population, (3) directional migration from the northern population to the southern population, and (4) directional migration from the southern population to the northern population. We used the same parameters to run all models and recoded the data as microsatellite repeat number from the original amplicon length so that we could use a model of Brownian motion to approximate the stepwise mutation of microsatellites (see migrate‐n manual). Prior distributions of θ (bounded at 0.0 and 150.0, window size of 15.0) and M (bounded at 0.0 and 50.0, window size of 5.0) were uniform. Mutation rate was calculated relative to the data. Each model was run for 50 million generations, sampling every 1,000 generations, with a burn‐in of 12.5 million. We used four variably heated chains following the methods of Beerli and Palczewski (2010). Convergence of the runs was determined from posterior probability distributions. To calculate Bayes factors, we used the log marginal likelihood provided by the Bezier approximation in the Migrate‐n output and followed recommendations in the program documentation to determine the probability of each model of migration.

### 2.8 Sequence data analysis

We downloaded 10 additional sequences from GenBank to supplement our dataset (*T. brasiliensis* from Brazil: KP134551, KP134552, KP134553; *T. brasiliensis* from the American Southwest: JF489129, JQ731812; *T. teniotis* from Iberia: DQ120907, DQ120908, DQ120909, DQ120910, EU360721). We performed two sets of tree reconstructions, one using only samples that had complete sequences (1,140 bp) and one with sequences trimmed (674 bp) to allow for incorporation of more individuals. Only 44 individuals of *T. brasiliensis* from The Bahamas (*n* = 40), Brazil (*n* = 3), and the American Southwest (*n* = 1) had complete sequences, so we will refer to this dataset as the intraspecific dataset. The dataset with trimmed sequences had 54 individuals of *T. brasiliensis* from The Bahamas (*n* = 44), Brazil (*n* = 3), and the American Southwest (*n* = 2), and *T. teniotis* from Iberia (*n* = 5), so we will refer to this dataset as the interspecific dataset. We used DnaSP v5 to calculate polymorphism of cytb sequences using only complete sequences of individuals from The Bahamas and Florida, because DnaSP cannot handle missing data (Librado & Rozas, 2009).

We used RAxML v8 (Stamatakis, 2014) for phylogeny reconstruction implemented in CIPRES Science Gateway (Miller, Pfeiffer, & Schwartz, 2010). Models of nuclear substitution were compared using Akaike information criterion corrected for small sample size (AICc) using jModelTest v2.1.7 (Darriba, Taboada, Doallo, & Posada, 2012) implemented in the CIPRES Science Gateway (Miller et al., 2010). The TrN+G model had the best AICc score for both datasets; however, this model cannot be implemented in RAxML, so we used GTR+G, which is the best model after TrN+G for both datasets. Instead of constraining an outgroup in the analyses, we rooted phylogenies in FigTree v1.4.2 (http://tree.bio.ed.ac.uk/software/figtree). The intraspecific dataset was rooted by the GenBank samples from Brazil, and the interspecific dataset was rooted by GenBank samples of *T. teniotis*. We used 1,000 bootstrap replicates to assess support at each node. We also used SplitsTree4 v4.14.2 (Huson & Bryant, 2006) to reconstruct a phylogenetic network of the intraspecific dataset using the NeighborNet method and 1,000 bootstrap replicates.

## 3 RESULTS

### 3.1 Population structure in the Bahamas

The nine microsatellites amplified in this study were found to be highly polymorphic in *Tadarida brasiliensis* from our study area, ranging from 8 to 46 alleles at each locus and averaging 23 alleles per locus. Florida and islands of the Little Bahama Bank share 96 private alleles not found in the Great Bahama Bank, 19 of which are present at a frequency ≥0.05. Islands of the Great Bahama Bank have 21 private alleles, of which two are present at a frequency ≥0.05.

Exact tests of the data separated by locality (all islands and Florida treated as separate populations) suggest that all loci in our study deviate from Hardy–Weinberg Equilibrium (HWE) at all island localities. After using sequential Bonferroni (Rice, 1989), four loci sampled from Long Island and Eleuthera (Great Bahama Bank) violate HWE and five loci sampled from Abaco and Grand Bahama (Little Bahama Bank) violate HWE. Exact tests indicate strong support (*p*‐value <.005) for heterozygote deficiency in the Grand Bahama population, but only 4–5 loci support deficiency in the Abaco, Eleuthera, and Long Island populations. There is no support for heterozygote deficiency for the Florida populations. Heterozygote excess is not supported by any of the loci at any locality. When sampling localities were grouped into two populations separated by the NPC, the northern population (Florida and the Little Bahama Bank) deviates from HWE at all, but two loci and the southern population (Great Bahama Bank) deviates in five of nine loci. Heterozygote deficiency is more strongly supported in the grouped dataset, especially for the northern population. In the northern population, eight or nine loci support heterozygote deficiency, and in the southern population, five of the nine loci support heterozygote deficiency. No loci in either population support heterozygote excess. Heterozygote deficiency may indicate that populations are experiencing the effects of genetic drift, nonrandom mating, or selection.

**Figure 3:**
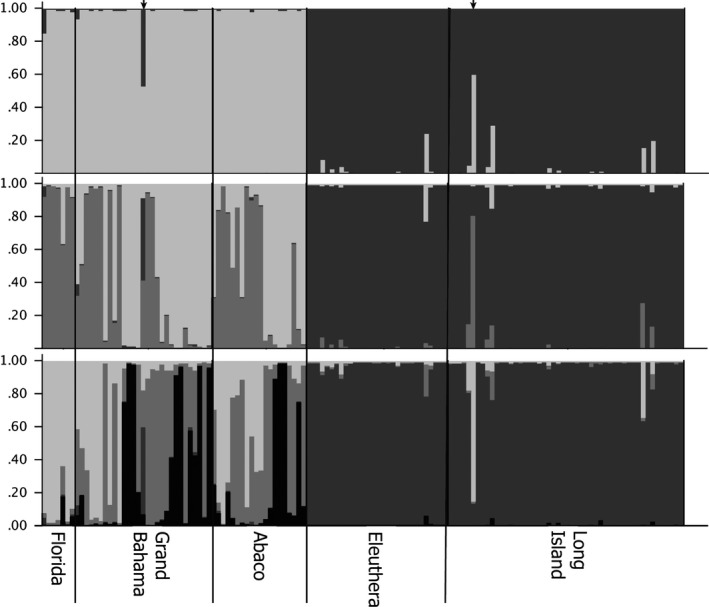
Structure plots under assumptions of *k* = 2 (top), *k* = 3 (middle), and *k* = 4 (bottom). Each vertical bar along the *x*‐axis represents the genotype of an individual, sorted by their sampling locality. The *y*‐axis indicates the proportion of the genotype that belongs to a given number of clusters (*k*). Each cluster is indicated with a different color. The two small arrows on top of the *k* = 2 plot indicate UF33074 (left) and UF31901 (right; see text for further information)

Analysis of the microsatellite data with Structure (Pritchard et al., 2000) and Structure Harvester (Evanno et al., 2005) indicates that an assumption of two populations is the best‐supported hypothesis given our data (Figure 3; Supporting Information). Under the assumption of two populations, Structure assigns individuals from the Great Bahama Bank into a population (southern population) distinct from individuals collected from the Little Bahama Bank and Florida (northern population; Figure 3). Structure indicated that two individuals (UF33074 and UF31901) may have migrant ancestry. UF33074 was sampled from Grand Bahama (north of the NPC), but 48% of its genotype matches the alleles in the Great Bahama Bank (south of the NPC). UF31901 was sampled from Long Island (south of the NPC), but 56% of its genotype matches the alleles in the northern population. If one assumes that there are three distinct populations (*k* = 3), Structure assigns approximately half of the individuals collected from the Little Bahama Bank to a new cluster, with the other individuals collected from the Little Bahama Bank remaining clustered with Florida (Figure 3). When the number of populations is set to 4, structure plots are the same as under the assumption of three populations with further subdivision within the Little Bahama Bank (Figure 3). This suggests that an assumption of *k* = 3 and *k* = 4 overestimates the number of clusters in this system.

Pairwise comparison of *F*_ST_ between the northern and southern populations suggests low differentiation (Table 1). However, pairwise *F*_ST_ estimates are significantly different than the level of differentiation expected under random permutations of the data (Table 1). *F*_ST_ cannot reach 1 when using highly variable loci, even when there are no shared alleles between two populations (Jost, 2008). *G*”_ST_ and Jost's *D*_EST_ are robust to high heterozygosity and more accurately characterize population differentiation when using microsatellite markers, which are highly variable (Meirmans & Hedrick, 2011). *G*”_ST_ and Jost's *D*_EST_ suggest high differentiation between the northern and southern populations (Table 1), in agreement with the assignment of individuals in our Structure analysis. *G*”_ST_ and *D*_EST_ provide evidence of differentiation between Florida and the Little Bahama, but not to the degree that the Little Bahama Bank is differentiated from the Great Bahama Bank (Table 2). We find no evidence of isolation by distance, because there is no significant correlation between geographic distance and genetic distance when compared to random.

**Table 1:** Pairwise genetic distance between banks of islands. Pairwise distances calculated in GenAlEx and DnaSP between northern and southern populations. Da (JC) and *D*_xy_ are pairwise sequence divergences. Significance is expressed either as a *p*‐value or as a 95% confidence interval. *p*‐values give the statistical significance of the data compared to differentiation expected from 999 random permutations of the data

Distance	Significance	Test
0.070	*p* = .001	*F*_ST_
0.057	*p* = .001	*G*_ST_
0.047	*p* = .0004	*R*_ST_
0.631	*p* = .001	*G*”_ST_
0.586	*p* = .001	Jost's D
0.047	0.024–0.069	Da (JC)
0.05	NA	*D*_xy_

**Table 2:** Pairwise *G*”_ST_ and Jost's D for islands. Pairwise distances calculated in GenAlEx between collecting localities. *G*”_ST_ values are below the diagonal and Jost's D is shown above the diagonal. Asterisks indicate differentiations that are significantly greater than expected by random (*p < .05, **p < .005)

Abaco	Grand Bahama	Florida	Eleuthera	Long Island	
–	0.035	**0.318	**0.639	**0.654	Abaco
0.038	–	**0.307	**0.598	**0.603	Grand Bahama
**0.342	**0.330	–	**0.448	**0.502	Florida
**0.682	**0.640	**0.501	–	*0.025	Eleuthera
**0.698	**0.649	**0.558	*0.034	–	Long Island

### 3.2 Estimates of dispersal

**Figure 4:**
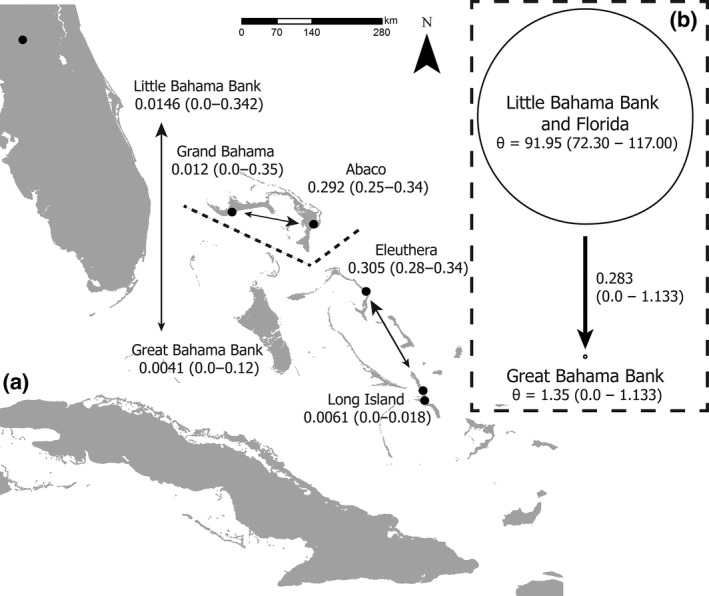
(a) BayesAss estimates of migration are provided on the map of The Bahamas (islands indicated in gray and sampling localities in black). The proportion of migrants is indicated under the island and bank labels, and black arrows indicate the pairs of islands for which gene flow was estimated. (b) Migrate‐n results are indicated on the right. The circles indicate relative population size, with the Great Bahama Bank having a much smaller population size than the Little Bahama Bank and Florida. Mutation‐scaled migration rate is shown along the black arrow

BayesAss suggests that within the last two generations, there has been no dispersal between the northern and southern populations in either direction (Florida individuals are not included in these analyses; Figure 4). The proportion of migrant individuals in the northern and southern populations (0.015 ± 0.0196 and 0.004 ± 0.008, respectively) is not significantly different from zero (Figure 4). Within each population (both northern and southern), there is evidence of recent directional dispersal between islands. Within the northern populations, more individuals have recently moved from Grand Bahama to Abaco than in the other direction (Figure 4). In the southern population, more individuals per generation have dispersed from Long Island to Eleuthera (Figure 4). Analysis of individual migrant ancestry in BayesAss confirms migrant ancestry of UF33074 (collected from Grand Bahama) suggested by Structure plots (Figure 3). There is a 0.990 probability that UF33074 is a first‐generation migrant from Long Island when each island is treated as a distinct population, and a 0.94 probability when individuals are grouped into the northern and southern populations. The other individual identified as a potential migrant in Structure analyses, UF31901 (collected from Long Island), was not found to be a recent migrant by BayesAss analysis. In the *k* = 3 Structure plots (Figure 3), UF31901 appears to share alleles with the individuals from Florida. We did not include any samples from Florida for our BayesAss analysis, which may explain why UF31901 does not appear as a migrant in this island‐only comparison.

Despite the clear lack of migration in the last two generations, as determined by BayesAss, Migrate‐n provides estimates of historic gene flow, because it assumes populations have reached mutation–migration–drift equilibrium. Using Bayes factors to compare four models of dispersal, we found that directional migration from the northern population to the southern population is the most strongly supported model (probability of 1.00). In this model of migration, Θ (4N_e_μ) of the northern population is significantly larger than that of the southern population (Figure 4). The mutation‐scaled immigration rate (*Μ* = *m*/μ, where *m* is the proportion of immigrants) from the Little Bahama Bank and Florida to the Great Bahama Bank was 0.283 (0.00–1.133; Figure 4). Using the mutation rate for mammalian microsatellites from Weber and Wong (1993), the rough migration rate from the north to the south is .00034 individuals per generation. Estimates of migration rate generated by migrate‐n may be incorrect when population sizes are large and the split between populations was very recent, because this scenario violates the assumption of mutation–migration–drift equilibrium (Russell, Goodman, Fiorentino, & Yoder, 2008; Whitlock & McCauley, 1999). BayesAss does not assume genetic equilibrium and supports migrate‐n results of low dispersal across the NPC. While BayesAss only measures contemporary gene flow, our estimate of low migration across the NPC is also supported by analyses of mitochondrial DNA and morphology (described below).

### 3.3 Mitochondrial phylogeography

The cytb data assembled for this study had 77 polymorphic sites and an average nucleotide diversity of 0.028. We found 17 unique haplotypes, 13 of which are represented by a single individual. There were three haplotypes that represented most individuals. The southern population haplotype had 16 individuals, a northern population haplotype had six individuals, and a haplotype specific to the Little Bahama Bank had three individuals. We found no haplotypes shared across the NPC. Mean pairwise sequence divergence is 0.047 between the northern and southern populations with three fixed mutations distinguishing these populations (Table 1). There was no differentiation between Abaco and Grand Bahama within the Little Bahama Bank, between islands of the Little Bahama Bank and Florida, or between Eleuthera and Long Island within the southern population.

**Figure 5:**
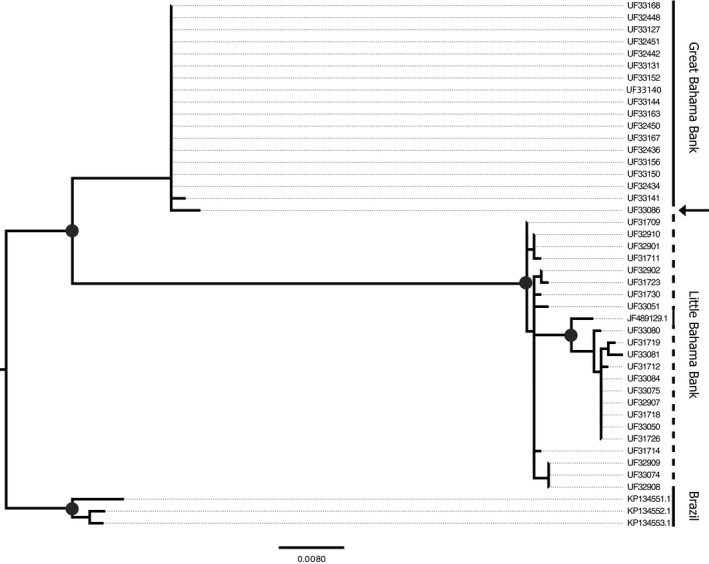
Intraspecific RAxML phylogeny with tip labels corresponding to Appendix S1. Nodes with greater than 90% bootstrap support are indicated with black dots. The arrow points to UF33086, which was collected from Grand Bahama in the Little Bahama Bank, but falls within the Great Bahama Bank clade. The dashed line indicates the individuals that were collected from the Little Bahama Bank, and the solid line within this dashed line indicates the single individual from the southwest USA (GenBank JF489129.1)

The intraspecific phylogeny, which only includes *T. brasiliensis*, indicates reciprocal monophyly of the northern population and the Great Bahama Bank (bootstrap support of 0.99), with the exception of a single individual, UF33086 (Figure 5). Reconstruction of a phylogenetic network gave the same results (Supporting Information). UF33086 was not identified as a migrant by Structure or BayesAss. The discrepancy between mitochondrial DNA and nuclear DNA (microsatellites) in UF33086 may be due to differences in the rate of mutation between mitochondrial DNA and microsatellites. UF33086 may have dispersed across the NPC further in the past than can be detected by rapidly mutating microsatellites. Despite evidence provided by our Structure and BayesAss analyses, UF33074 (collected from Grand Bahama) fell within the clade containing other samples from the northern population (Figure 5). Differences in the rate of mutation between mitochondrial DNA and microsatellites may also explain why UF33074 was identified as a recent migrant using microsatellites, but does not fall within the southern clade in phylogenetic analyses. UF31901, also identified by Structure as a potential migrant, was not sequenced (Appendix S1). There is no supported structure within the northern and southern clades (Figure 5).

**Figure 6:**
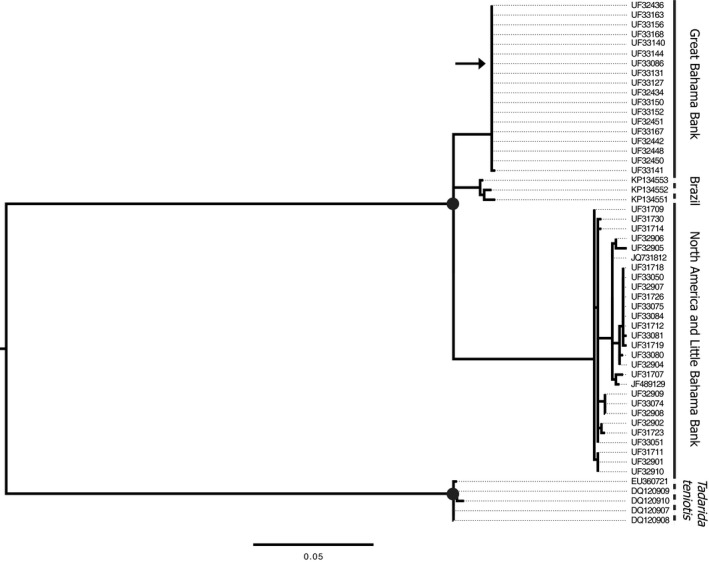
Interspecific RAxML phylogeny with tip labels corresponding to Appendix S1. Nodes with greater than 90% bootstrap support are indicated by black dots. The node joining the Great Bahama Bank clade and the clade containing North America, the Little Bahama Bank and Brazil is strongly supported with a bootstrap of 100. The node joining the Brazil clade to the North America and Little Bahama Bank clade is not supported, with a bootstrap of 34. The arrow points to UF33086, which was collected from the Little Bahama Bank

The interspecific phylogeny, which includes *T. brasiliensis* from the mainland and *T. teniotis*, also supports reciprocal monophyly between the northern and southern populations, with the exception of UF33086 (Figure 6). The clade containing Brazilian *T. brasiliensis* may be more closely related to the northern population and North American samples than to the southern population, but their position in the phylogeny is not well supported (bootstrap of 0.39; Figure 6).

### 3.4 Morphological differentiation

**Figure 7:**
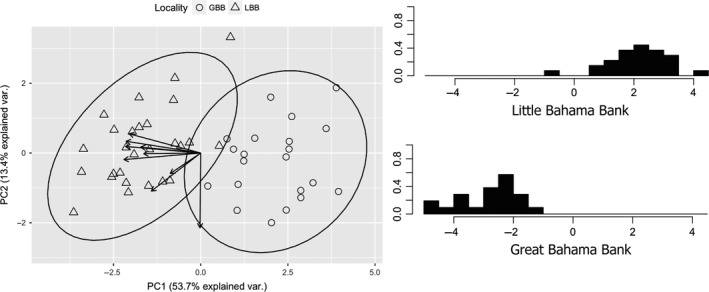
Principal components analysis (left) indicates that the Great Bahama Bank (GBB) and Little Bahama Bank (LBB) individuals tend to be different from each other. Vectors pointing toward the PC2 axis represent skull measurements, and the single vector pointing to the PC1 axis is the forearm measurement. Discriminant function analysis (right) also shows differentiation between the Great and Little Bahama Bank, with the exception of one individual from the Little Bahama Bank

Analyses of the male‐only dataset and the combined (male + female) dataset produced similar results, so we present results for the combined dataset. Principal components analysis (PCA) indicated that individuals from the Little Bahama Bank and Florida are not distinguishable from one another using our characters. The NPC is the notable divide between morphologically distinct groupings to the north and south. Principal component (PC) 1 explains 54% of the variation and primarily represents skull measurements (Figure 7). PC2 explains 13% of the variation and primarily represents forearm length (Figure 7). Linear discriminant analysis (LDA) assigned individuals correctly to the northern and southern populations 96% of the time. Only 1 individual (UF32202, a female collected from Grand Bahama) was incorrectly identified using LDA (Figure 7). UF32202 was not sequenced, but microsatellites do not indicate migrant ancestry of this individual. This may be due to sexual dimorphism, but we did not have enough skulls of female bats to measure differences between the sexes.

## 4 DISCUSSION AND CONCLUSIONS

We investigated the population connectivity among and between islands of the Little and Great Bahama Banks using microsatellites, mitochondrial DNA, and morphology. We found a major break in the population structure coinciding with the NPC, which separates the Little Bahama Bank from the Great Bahama Bank. Populations of two other species, *Macrotus waterhousii* and *Erophylla sezekorni*, are similarly distinct across the NPC (Muscarella et al., 2011). We expected *T. brasiliensis* to show low population differentiation across the NPC, due to its high dispersal ability compared to bats with low and slow flight patterns, like *M. waterhousii* and *E. sezekorni*. However, our findings suggest that there is high population differentiation and low gene flow across the NPC in *T. brasiliensis*.

Microsatellite data identified two distinct populations of *T. brasiliensis* in The Bahamas, separated by the NPC. Phylogenetic reconstruction based on cytb confirmed our findings based on microsatellite data that the northern and southern populations are separated into well‐supported clades (Figures 5 and 6). Pairwise nucleotide diversity between the northern and southern populations (~5%) is high and may suggest that this divergence is not recent. Lastly, cranial and forearm measurements also show differentiation between the northern and southern populations. *Tadarida brasiliensis* populations on the north and south sides of the NPC are distinct genetically and morphologically.

Previous research on bat dispersal in island systems has detected species‐specific patterns, with some species showing high population connectivity, while others instead exhibit low connectivity and high divergence (Carstens et al., 2004; Fleming et al., 2010; Muscarella et al., 2011; Salgueiro et al., 2004). Phylogeographic studies of bats in the Caribbean have previously examined only phyllostomid species. *Artibeus jamaicensis*,* Brachyphylla cavernarum*,* Erophylla bombifrons*, and *Erophylla sezekorni* all show population connectivity across Caribbean islands (although, within The Bahamas, *E. sezekorni* exhibits structuring across the NPC). *Macrotus waterhousii* and *Ardops nichollsi* show considerable population structuring (Carstens et al., 2004; Fleming et al., 2010; Muscarella et al., 2011). Of these studies, only Muscarella et al. (2011) included samples from The Bahamas that permitted assessment of the effects of the NPC on bat population connectivity. Our data show *T. brasiliensis* has a similar pattern of population connectivity as *E. sezekorni*, with both species exhibiting multi‐island population clusters in The Bahamas that are distinct across the NPC (Muscarella et al., 2011). *Tadarida brasiliensis* and *E. sezekorni* both exhibit a higher degree of population connectivity than *M. waterhousii* in The Bahamas (Muscarella et al., 2011).

Unlike *E. sezekorni*, we found no evidence of isolation by distance in *T. brasiliensis* (Muscarella et al., 2011). Despite having distinct populations separated by the NPC that show little to no evidence of contemporary dispersal, the long‐term estimated migration rate for *E. sezekorni* and *M. waterhousii* is higher across the NPC than in *T. brasiliensis* (Muscarella et al., 2011). Divergence dates between populations separated by the NPC in *E. sezekorni* (40 kya) and *M. waterhousii* (15 kya) may be more recent than in *T. brasiliensis* (assuming 5% sequence divergence and 0.05 substitutions per site per million years; Nabholz, Glémin, & Galtier, 2008). This raises the possibility that patterns detected in our analysis may be the result of species‐level differentiation between the northern and southern populations.

Cytb data suggest that individuals of *T. brasiliensis* from Brazil form a distinct clade that may be more closely related to mainland North American and northern Bahamas bats than to those in the southern Bahamas, which form a distinct clade. Previous research has found no genetic differentiation of *T. brasiliensis* across its North and Central American range, but *T. b. brasiliensis*, the South American subspecies, is distinct from its North and Central American conspecifics (Morales et al., 2016; Russell & McCracken, 2006). If the Great Bahama Bank and other Caribbean islands were colonized from South America, this might explain the level of differentiation we see across the NPC. The position of Brazilian samples in our cytb phylogeny is unclear, but is currently clustered with the North American and northern Bahamas samples, which contradicts the hypothesis of South American colonization of the southern Bahamas. Even in the scenario where the NPC is the meeting place of the leading edges of two species in the genus *Tadarida* and not the result of in situ divergence of two populations due to the difficulty of dispersing across the NPC, the NPC is still a barrier to dispersal in bats. Our BayesAss analyses indicate little to no recent dispersal.

The results of our phylogeographic analyses of cytb data including samples from Brazil and mainland North America suggest that the Great Bahama Bank population (southern population) might represent a cryptic species of the genus *Tadarida* that is closely related to *T. brasiliensis*. Such a hypothesis is consistent with the evidence for low levels of recent migration, historical directional dispersal from the Little Bahama Bank to the Great Bahama Bank, high levels of population differentiation, and lack of morphological overlap between Great Bahama Bank *Tadarida* and the northern population sampled from the Little Bahama Bank and Florida. It has previously been suggested based on limited morphological sampling that Caribbean populations of *Tadarida brasiliensis* are distinct from those on the mainland and that individuals in The Bahamas represent a unique subspecies—*T. b. bahamensis* (type specimen collected from Governor's Harbor, Eleuthera, and preserved at the University of Pennsylvania; Rehn, 1902; Shamel, 1931; Schwartz, 1955). Future studies should include better sampling of other islands in the Caribbean and mainland individuals, especially from South America, to better assess the possibility of a Caribbean species in the genus *Tadarida*.

## CONFLICT OF INTEREST

None declared.
