# Supplementary material for: Population structure of a widespread bat (Tadarida brasiliensis) in an island system
Source: Ecol Evol. 2017 Aug 17;7(19):7585–98. doi: 10.1002/ece3.3233 (PMC5632666; doi:10.1002/ece3.3233)

Supplemental Information: Population structure of a widespread bat (*Tadarida brasiliensis*) in an island system

Materials and Methods – For gel electrophoresis, 2μL of extraction volume or PCR product were mixed with 4μL 1x loading dye (diluted from 6x concentration; blue/orange loading dye, Promega, Madison, Wisconsin) and loaded into 1% agarose gels (1g agarose for 100mL TAE buffer; Thermo Fisher Scientific, Fair Lawn, New Jersey) and electrophoresed along with 1kb ladder (extractions and gene-targeted PCR products; Apex Scientific, Stony Brook, New York) or 100kb ladder (microsatellite PCR products; New England Biolabs, Inc., Ipswich, Massachusetts). Gels were stained with Ethidium Bromide to visualize DNA.

Figure S1 – Graph of mean ln probability of L(K) generated from Structure analyses. See text for details on the Structure parameters.

Figure S2 – Phylogenetic network reconstructed in SplitsTree using the intraspecific dataset.


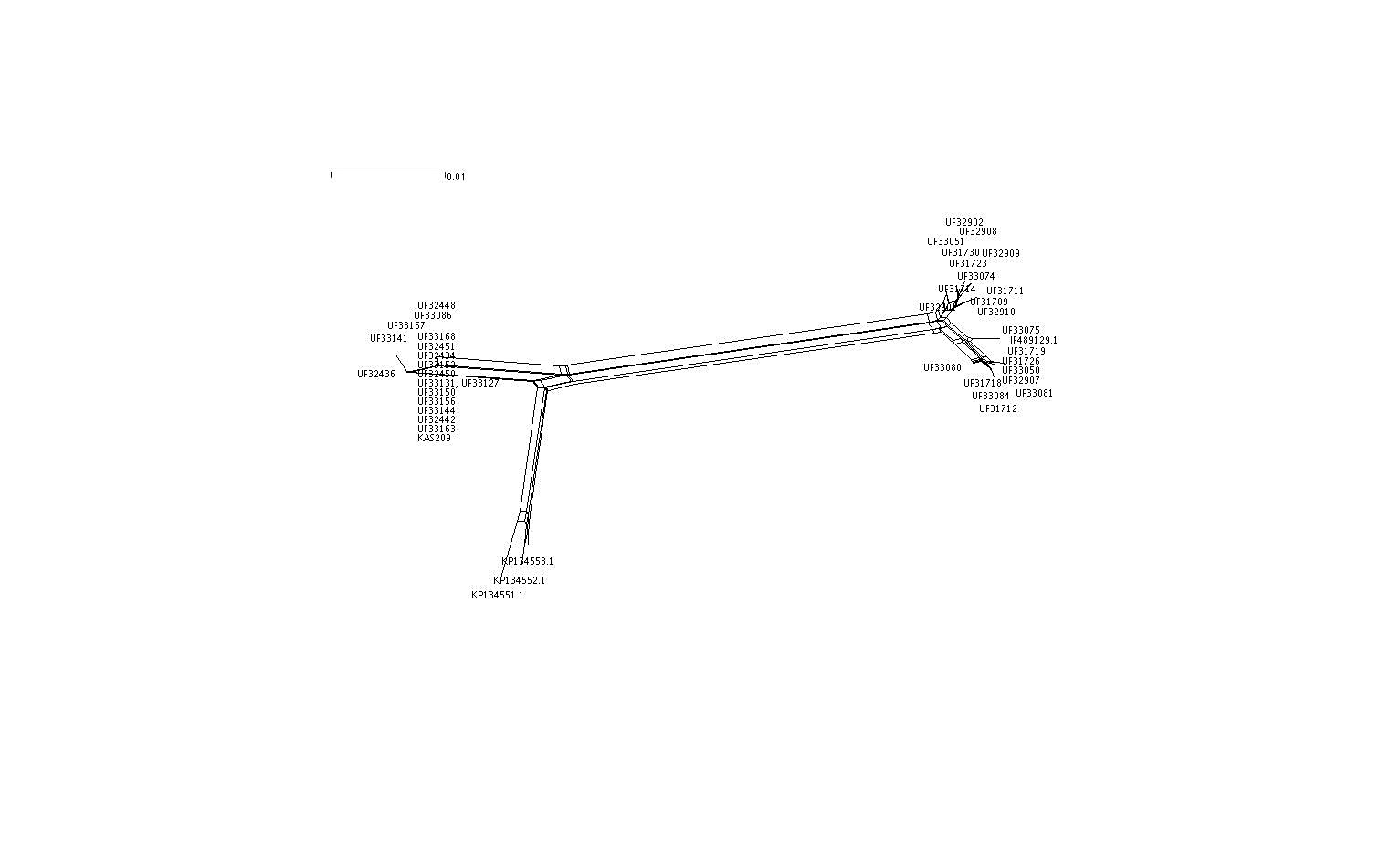

Supplement: Supplementary file 1 [file ECE3-7-7585-s001.docx]
