# Supplementary material for: Population structure of a widespread bat (Tadarida brasiliensis) in an island system
Source: Ecol Evol. 2017 Aug 17;7(19):7585–98. doi: 10.1002/ece3.3233 (PMC5632666; doi:10.1002/ece3.3233)
Supplement: Supplementary file 2 [file ECE3-7-7585-s002.docx]

Appendix 1. Table of the individuals used in this study, including the Florida Museum of Natural History (FLMNH) identification number, locality of collection, sex, types of data collected, and whether there is a voucher specimen associated with the catalogue number. Where there are check marks in the microsatellite and cytb columns and also a voucher specimen associated with the catalogue number, organ tissue was used in the genetic analysis. In all other cases, wing punches were used.

| **FLMNH** | **Locality** | **Sex** | **Microsat.** | **CytB** | **Morphology** | **Notes** |
| --- | --- | --- | --- | --- | --- | --- |
| 264 | Alachua | male |  |  | ✓ | Vouchered |
| 267 | Alachua | female |  |  | ✓ | Vouchered |
| 10677 | Alachua | female |  |  | ✓ | Vouchered |
| 10696 | Alachua | male |  |  | ✓ | Vouchered |
| 10854 | Alachua | female |  |  | ✓ | Vouchered |
| 10905 | Alachua | female |  |  | ✓ | Vouchered |
| 10935 | Alachua | male |  |  | ✓ | Vouchered |
| 11194 | Alachua | male |  |  | ✓ | Vouchered |
| 11198 | Alachua | female |  |  | ✓ | Vouchered |
| 11320 | Alachua | male |  |  | ✓ | Vouchered |
| 11338 | Alachua | male |  |  | ✓ | Vouchered |
| 11351 | Alachua | female |  |  | ✓ | Vouchered |
| 11404 | Alachua | male |  |  | ✓ | Vouchered |
| 11421 | Alachua | female |  |  | ✓ | Vouchered |
| 11449 | Alachua | male |  |  | ✓ | Vouchered |
| 24867 | Abaco | female |  |  | ✓ | Vouchered |
| 24868 | Abaco | male |  |  | ✓ | Vouchered |
| 28300 | Alachua | female |  |  | ✓ | Vouchered |
| 28311 | Alachua | male |  |  | ✓ | Vouchered |
| 28314 | Alachua | male |  |  | ✓ | Vouchered |
| 28331 | Alachua | male |  |  | ✓ | Vouchered |
| 31678 | Alachua | female |  |  | ✓ | Vouchered |
| 31707 | Alachua | male |  | MF135770 |  | Vouchered |
| 31709 | Alachua | female | ✓ | MF135771 |  | Vouchered |
| 31711 | Alachua | female | ✓ | MF135772 |  | Vouchered |
| 31712 | Alachua | not recorded | ✓ | MF135773 |  | Vouchered |
| 31714 | Alachua | male | ✓ | MF135774 |  | Vouchered |
| 31718 | Alachua | male | ✓ | MF135775 |  | Vouchered |
| 31719 | Alachua | female | ✓ | MF135776 |  | Vouchered |
| 31723 | Alachua | female | ✓ | MF135777 |  | Vouchered |
| 31726 | Alachua | male |  | MF135778 |  | Vouchered |
| 31730 | Alachua | male |  | MF135779 |  | Vouchered |
| 31788 | Eleuthera | male |  |  | ✓ | Vouchered |
| 31789 | Eleuthera | male |  |  | ✓ | Vouchered |
| 31790 | Eleuthera | male |  |  | ✓ | Vouchered |
| Appendix 1 Continued. | | | | | | |
| **FLMNH** | **Locality** | **Sex** | **Microsat.** | **CytB** | **Morphology** | **Notes** |
| 31791 | Eleuthera | male |  |  | ✓ | Vouchered |
| 31792 | Eleuthera | male |  |  | ✓ | Vouchered |
| 31793 | Eleuthera | male | ✓ |  | ✓ | Vouchered |
| 31794 | Eleuthera | male | ✓ |  | ✓ | Vouchered |
| 31795 | Eleuthera | male | ✓ |  | ✓ | Vouchered |
| 31796 | Eleuthera | male | ✓ |  | ✓ | Vouchered |
| 31797 | Eleuthera | male | ✓ |  | ✓ | Vouchered |
| 31798 | Eleuthera | male | ✓ |  | ✓ | Vouchered |
| 31799 | Eleuthera | male | ✓ |  | ✓ | Vouchered |
| 31800 | Eleuthera | male | ✓ |  | ✓ | Vouchered |
| 31801 | Eleuthera | male | ✓ |  | ✓ | Vouchered |
| 31802 | Eleuthera | male | ✓ |  | ✓ | Vouchered |
| 31803 | Eleuthera | male |  |  | ✓ | Vouchered |
| 31804 | Eleuthera | female |  |  | ✓ | Vouchered |
| 31805 | Eleuthera | female |  |  | ✓ | Vouchered |
| 31806 | Eleuthera | female |  |  | ✓ | Vouchered |
| 31807 | Eleuthera | female |  |  | ✓ | Vouchered |
| 31881 | Long Island | male | ✓ |  | ✓ | Vouchered |
| 31882 | Long Island | male | ✓ |  | ✓ | Vouchered |
| 31883 | Long Island | male | ✓ |  | ✓ | Vouchered |
| 31884 | Long Island | female | ✓ |  | ✓ | Vouchered |
| 31885 | Long Island | male | ✓ |  | ✓ | Vouchered |
| 31901 | Long Island | male | ✓ |  | ✓ | Vouchered |
| 31902 | Long Island | female | ✓ |  | ✓ | Vouchered |
| 31903 | Long Island | male | ✓ |  | ✓ | Vouchered |
| 31904 | Long Island | male | ✓ |  | ✓ | Vouchered |
| 31905 | Long Island | not recorded | ✓ |  | ✓ | Vouchered |
| 32118 | Abaco | female | ✓ |  | ✓ | Vouchered |
| 32119 | Abaco | female | ✓ |  |  |  |
| 32120 | Abaco | male | ✓ |  | ✓ | Vouchered |
| 32121 | Abaco | female | ✓ |  |  |  |
| 32122 | Abaco | female | ✓ |  |  |  |
| 32123 | Abaco | female | ✓ |  |  |  |
| 32124 | Abaco | female | ✓ |  |  |  |
| 32125 | Abaco | female | ✓ |  |  |  |
| 32126 | Abaco | female | ✓ |  |  |  |
| 32127 | Abaco | female | ✓ |  |  |  |
| 32197 | Grand Bahama | male |  |  | ✓ | Vouchered |
| 32198 | Grand Bahama | male | ✓ |  | ✓ | Vouchered |
| 32199 | Grand Bahama | male | ✓ |  | ✓ | Vouchered |
| 32200 | Grand Bahama | female | ✓ |  | ✓ | Vouchered |
| 32201 | Grand Bahama | male | ✓ |  | ✓ | Vouchered |
| 32202 | Grand Bahama | female | ✓ |  | ✓ | Vouchered |
| 32203 | Grand Bahama | male | ✓ |  | ✓ | Vouchered |
| 32204 | Grand Bahama | female | ✓ |  | ✓ | Vouchered |
| 32205 | Grand Bahama | male | ✓ |  | ✓ | Vouchered |
| 32206 | Grand Bahama | male | ✓ |  | ✓ | Vouchered |
| Appendix 1 Continued. | | | | | | |
| **FLMNH** | **Locality** | **Sex** | **Microsat.** | **CytB** | **Morphology** | **Notes** |
| 32207 | Grand Bahama | male | ✓ |  | ✓ | Vouchered |
| 32433 | Eleuthera | male | ✓ |  |  |  |
| 32434 | Eleuthera | not recorded | ✓ | MF135745 |  |  |
| 32435 | Eleuthera | male | ✓ |  |  |  |
| 32436 | Eleuthera | male | ✓ | MF135746 |  |  |
| 32437 | Eleuthera | male | ✓ |  |  |  |
| 32438 | Eleuthera | male | ✓ |  |  |  |
| 32439 | Eleuthera | male | ✓ |  |  |  |
| 32440 | Eleuthera | male | ✓ |  |  |  |
| 32441 | Eleuthera | male | ✓ |  |  |  |
| 32442 | Eleuthera | male | ✓ | MF135747 |  |  |
| 32443 | Eleuthera | male | ✓ |  |  |  |
| 32444 | Eleuthera | male | ✓ |  |  |  |
| 32445 | Eleuthera | male | ✓ |  |  |  |
| 32446 | Eleuthera | male | ✓ |  |  |  |
| 32447 | Eleuthera | male | ✓ |  |  |  |
| 32448 | Eleuthera | male | ✓ | MF135748 |  |  |
| 32449 | Eleuthera | male | ✓ |  |  |  |
| 32450 | Eleuthera | male | ✓ | MF135749 |  |  |
| 32451 | Eleuthera | female | ✓ | MF135750 |  |  |
| 32452 | Eleuthera | male | ✓ |  |  |  |
| 32901 | Abaco | male | ✓ | MF135735 |  |  |
| 32902 | Abaco | male | ✓ | MF135736 |  |  |
| 32903 | Abaco | male | ✓ | MF135737 |  |  |
| 32904 | Abaco | male | ✓ | MF135738 |  |  |
| 32905 | Abaco | female | ✓ | MF135739 |  |  |
| 32906 | Abaco | female | ✓ | MF135740 |  |  |
| 32907 | Abaco | female | ✓ | MF135741 |  |  |
| 32908 | Abaco | male | ✓ | MF135742 |  |  |
| 32909 | Abaco | female | ✓ | MF135743 |  |  |
| 32910 | Abaco | female | ✓ | MF135744 |  |  |
| 33050 | Grand Bahama | female | ✓ | MF135751 |  |  |
| 33051 | Grand Bahama | female | ✓ | MF135752 |  |  |
| 33052 | Grand Bahama | female | ✓ |  |  |  |
| 33073 | Grand Bahama | male | ✓ |  |  |  |
| 33074 | Grand Bahama | female | ✓ | MF135753 |  |  |
| 33075 | Grand Bahama | female | ✓ | MF135754 |  |  |
| 33076 | Grand Bahama | female | ✓ |  |  |  |
| 33077 | Grand Bahama | male | ✓ |  |  |  |
| 33078 | Grand Bahama | male | ✓ |  |  |  |
| 33079 | Grand Bahama | female | ✓ |  |  |  |
| 33080 | Grand Bahama | female | ✓ | MF135755 |  |  |
| 33081 | Grand Bahama | male | ✓ | MF135756 |  |  |
| 33082 | Grand Bahama | female | ✓ |  |  |  |
| 33083 | Grand Bahama | female | ✓ |  |  |  |
| 33084 | Grand Bahama | female | ✓ | MF135757 |  |  |
| 33085 | Grand Bahama | male | ✓ |  |  |  |
| Appendix 1 Continued. | | | | | | |
| **FLMNH** | **Locality** | **Sex** | **Microsat.** | **CytB** | **Morphology** | **Notes** |
| 33086 | Grand Bahama | female | ✓ | MF135758 |  |  |
| 33087 | Grand Bahama | female | ✓ |  |  |  |
| 33088 | Grand Bahama | female | ✓ |  |  |  |
| 33127 | Long Island | male | ✓ | MF135759 |  |  |
| 33128 | Long Island | male | ✓ |  |  |  |
| 33129 | Long Island | male | ✓ |  |  |  |
| 33130 | Long Island | male | ✓ |  |  |  |
| 33131 | Long Island | male | ✓ | MF135760 |  |  |
| 33132 | Long Island | male | ✓ |  |  |  |
| 33133 | Long Island | male | ✓ |  |  |  |
| 33134 | Long Island | male | ✓ |  |  |  |
| 33135 | Long Island | male | ✓ |  |  |  |
| 33136 | Long Island | male | ✓ |  |  |  |
| 33137 | Long Island | male | ✓ |  |  |  |
| 33138 | Long Island | male | ✓ |  |  |  |
| 33139 | Long Island | male | ✓ |  |  |  |
| 33140 | Long Island | male | ✓ | MF135761 |  |  |
| 33141 | Long Island | male | ✓ | MF135762 |  |  |
| 33142 | Long Island | male | ✓ |  |  |  |
| 33143 | Long Island | male | ✓ |  |  |  |
| 33144 | Long Island | male | ✓ | MF135763 |  |  |
| 33145 | Long Island | male | ✓ |  |  |  |
| 33146 | Long Island | male | ✓ |  |  |  |
| 33149 | Long Island | male | ✓ |  |  |  |
| 33150 | Long Island | male | ✓ | MF135764 |  |  |
| 33151 | Long Island | female | ✓ |  |  |  |
| 33152 | Long Island | female | ✓ | MF135765 |  |  |
| 33153 | Long Island | female | ✓ |  |  |  |
| 33154 | Long Island | female | ✓ |  |  |  |
| 33155 | Long Island | female | ✓ |  |  |  |
| 33156 | Long Island | female | ✓ | MF135766 |  |  |
| 33157 | Long Island | male | ✓ |  |  |  |
| 33158 | Long Island | female | ✓ |  |  |  |
| 33159 | Long Island | female | ✓ |  |  |  |
| 33160 | Long Island | female | ✓ |  |  |  |
| 33161 | Long Island | female | ✓ |  |  |  |
| 33162 | Long Island | female | ✓ |  |  |  |
| 33163 | Long Island | not recorded | ✓ | MF135767 |  |  |
| 33164 | Long Island | female | ✓ |  |  |  |
| 33165 | Long Island | female | ✓ |  |  |  |
| 33166 | Long Island | female | ✓ |  |  |  |
| 33167 | Long Island | female | ✓ | MF135768 |  |  |
| 33168 | Long Island | female | ✓ | MF135769 |  |  |
